# A Plant Biologist’s Toolbox to Study Translation

**DOI:** 10.3389/fpls.2018.00873

**Published:** 2018-07-02

**Authors:** Serina M. Mazzoni-Putman, Anna N. Stepanova

**Affiliations:** Department of Plant and Microbial Biology, Program in Genetics, North Carolina State University, Raleigh, NC, United States

**Keywords:** translation, ribosome, Ribo-seq, TRAP, polysome profiling, toeprinting, reporter, amino acid tagging

## Abstract

Across a broad range of species and biological questions, more and more studies are incorporating translation data to better assess how gene regulation occurs at the level of protein synthesis. The inclusion of translation data improves upon, and has been shown to be more accurate than, transcriptional studies alone. However, there are many different techniques available to measure translation and it can be difficult, especially for young or aspiring scientists, to determine which methods are best applied in specific situations. We have assembled this review in order to enhance the understanding and promote the utilization of translational methods in plant biology. We cover a broad range of methods to measure changes in global translation (e.g., radiolabeling, polysome profiling, or puromycylation), translation of single genes (e.g., fluorescent reporter constructs, toeprinting, or ribosome density mapping), sequencing-based methods to uncover the entire translatome (e.g., Ribo-seq or translating ribosome affinity purification), and mass spectrometry-based methods to identify changes in the proteome (e.g., stable isotope labeling by amino acids in cell culture or bioorthogonal noncanonical amino acid tagging). The benefits and limitations of each method are discussed with a particular note of how applications from other model systems might be extended for use in plants. In order to make this burgeoning field more accessible to students and newer scientists, our review includes an extensive glossary to define key terms.

## Introduction

How do scientists go about understanding the complex genetic regulation that occurs in a cell? In many cases, it is not the gene itself that is of interest, but rather the functional product of that gene and the cellular and biological processes in which it is involved. Often, that functional product is a protein, but there are numerous regulatory steps between a gene and the protein it encodes. For many decades, changes in transcription have been used as a surrogate measure for changes in protein. However, it is well established that changes in mRNA do not paint an accurate picture of what is happening to protein levels (de Sousa Abreu et al., 2009; Maier et al., 2009; Rajasundaram et al., 2014).

Ideally, gene expression would be studied by quantifying changes in functional proteins, but the quantification of specific proteins can be difficult to achieve, especially when studying plants. Unlike yeast or mammalian systems, for many plant species there are no cell lines or readily available *in vitro* systems, and creating transgenics may be difficult or impossible. Plants present another challenge in that samples often come from a “natural” setting, rather than the laboratory (compared to engineered cell lines, animal models, etc.). The incorporation of a label, sample manipulation to introduce plasmids, and other modifications are not feasible in these situations. The ability to take samples directly from the field or forest to the lab is a mandate for many plant researchers. Additionally, there are very few reliable antibodies for the detection of endogenous plant proteins. An alternative approach to looking at proteins is to study translation. Because protein synthesis is a very energy-expensive process (Jewett et al., 2009), it is reasonable to expect that proteins will only be produced when needed, and therefore, a good correlation between translation rates and protein levels is predicted. In fact, a much better correlation between translation rate and protein levels (than between mRNA abundance and protein levels) has been demonstrated (Ingolia et al., 2009). Measuring translation allows researchers to identify not just which transcripts are present, but which transcripts are being made into proteins. Moreover, an increasing number of reports are finding that regulation of specific genes at the level of translation is critical to many plant cellular processes (Merchante et al., 2017) and that features in the transcript itself can regulate translation (von Arnim et al., 2014).

In this review, we discuss the methods that have been employed for evaluating translation in plants, reflect on the methods used in other systems and whether they can be extended to plants, and highlight areas for improving the use of translation as a measure of gene expression. The techniques are presented according to the molecule or molecular interaction studied: first, we address methods for quantifying new protein synthesis; next, those that measure ribosome:mRNA interactions as a surrogate for translation; then, methods that allow for live imaging of translation; and finally, methods that detect co-translational mRNA decay. For easier visualization and reference, all major techniques are also graphically represented in cartoon schematics and summarized in a table (see **Table 1**) with example publications cited. We chose to tailor this discussion to graduate students and other beginning scientists with the goal of making the study of translation more accessible and, therefore, more appealing to young researchers. At the end of this review, we have included a glossary of all underlined terms discussed in this article for easy cross-reference.

**Table 1:** Summary of the techniques discussed in this review.

Technique	Overview of rationale	What is measured	Selected references
Radiolabeling	Newly synthesized proteins incorporate radiolabeled amino acids.	Radioactive emission from total protein reflects the translation status of the sample.	Lageix et al., 2008; Galland et al., 2014; Wang et al., 2017
FUNCAT	Newly synthesized proteins incorporate a non-canonical amino acid that can be detected by “click” chemistry.	Fluorescence from a “click” chemistry reaction reflects the translation status of the sample.	Tom Dieck et al., 2012; Glenn et al., 2017
Cell-free protein expression system	The conditions for translation can be tightly controlled and exogenous elements easily introduced in a cell-free system.	The abundance of the protein of interest is examined under customizable conditions.	Murota et al., 2011; Buntru et al., 2014; Alvarez et al., 2016
Translational reporter fusion	A reporter gene that is easily detected at the protein level by fluorescence, antibodies, etc., is fused to a gene of interest.	The abundance of a reporter protein is detected as a surrogate for the protein of interest.	Tabuchi et al., 2006; Eastmond et al., 2010; Paik et al., 2012
SILAC	A pulse of isotope-labeled amino acids specifically marks newly synthesized proteins.	The presence of an isotope distinguishes new proteins upon MS analysis.	Ong et al., 2002; Gruhler et al., 2005; Schwanhausser et al., 2009; Lewandowska et al., 2013
BONCAT	Newly synthesized proteins incorporate a non-canonical amino acid that can be isolated by “click” chemistry.	MS analysis identifies affinity-purified proteins.	Glenn et al., 2017
QuanCAT	A pulse of non-canonical amino acids specifically marks newly synthesized proteins that can be isolated by “click” chemistry.	MS analysis identifies affinity-purified, newly synthesized proteins.	Howden et al., 2013
Puromycylation	Puromycin “tags” newly synthesized proteins.	Puromycin incorporation serves as a surrogate for global translation.	Basbouss-Serhal et al., 2015
SunSeT	Newly synthesized proteins incorporate puromycin, which can be detected with antibodies.	Puromycin incorporation serves as a surrogate for translation.	Schmidt et al., 2009; Van Hoewyk, 2016
RPM	Puromycin “tags” newly synthesized proteins and a chemical locks ribosomes in place.	Puromycin detection reveals the location of nascent peptides.	Seedhom et al., 2016
PUNCH-P	Biotinylated puromycin “tags” nascent peptides for isolation with streptavidin beads.	Purified proteins are analyzed by MS.	Aviner et al., 2013
Polysome profiling	Ribosome:mRNA complexes are separated using ultracentrifugation through a sucrose gradient.	The distribution of ribosomes shows global translation trends; the abundance of transcripts in different fractions is detected by qPCR, sequencing, etc.	Kawaguchi et al., 2004; Mustroph et al., 2009a; Karginov and Hannon, 2013; Yanguez et al., 2013; Layat et al., 2014; Basbouss-Serhal et al., 2015; Bai et al., 2017
RDM	The number of ribosomes on a transcript alters the sedimentation rate of the transcript.	Ribosome density is deduced from the size and fractionation distribution of mRNA fragments.	Arava et al., 2005
Toeprinting	A ribosome present on a transcript blocks RT and produces truncated cDNA products.	The length of RT products indicates the presence or absence of ribosomes on a transcript.	Anthony and Merrick, 1992; Gould et al., 2005; Hayashi et al., 2017
Ribosome footprinting (Ribo-seq)	A ribosome present on a transcript protects the transcript from RNase digestion, leaving behind ribosome “footprints”.	Ribosome footprints are sequenced to reveal the location and average density of ribosomes across the transcriptome.	Liu et al., 2013; Juntawong et al., 2014; Lei et al., 2015; Li et al., 2015; Merchante et al., 2015; Chotewutmontri and Barkan, 2016; Hsu et al., 2016; Lukoszek et al., 2016; Bazin et al., 2017; Xu et al., 2017; Zoschke et al., 2017
TCP-seq	Crosslinking of ribosomes to transcripts allows detection of ribosome subunit:mRNA interactions.	Ribosome footprints from small subunit and monosome fractions are sequenced to reveal translation dynamics.	Archer et al., 2016
TRAP, TRAP-seq	Tagged ribosomes with associated transcripts are affinity purified from cellular lysate.	The sequence of ribosome-bound transcripts identifies the translatome.	Zanetti et al., 2005; Mustroph et al., 2009b; Jiao and Meyerowitz, 2010; Rajasundaram et al., 2014; Wang and Jiao, 2014; Vragovic et al., 2015
Gaussia luciferase	“Flash” kinetics allow for live visualization of newly synthesized protein of interest.	Bioluminescence acts as a marker of tagged, newly synthesized proteins.	Na et al., 2016
Photoswitchable protein	Exposure to UV light changes the emission spectrum of tagged protein of interest.	Fluorescent emission in the original spectrum acts as a surrogate for newly synthesized proteins.	Leung et al., 2006; Raab-Graham et al., 2006; Leung and Holt, 2008; Vogelaar et al., 2009
Ribosome “knock-off”	An advancing ribosome knocks the fluorescent, hairpin-specific RNA binding protein off of the transcript of interest.	A change is fluorescent signal localization acts as an indicator of active translation.	Halstead et al., 2015
Fluorescent colocalization	A fluorescently marked transcript co-localizes with a fluorescently marked ribosome or nascent peptide.	A change in fluorescent signal localization or new fluorescent foci acts as an indicator of active translation.	Katz et al., 2016; Morisaki et al., 2016; Wang et al., 2016; Wu et al., 2016; Yan et al., 2016
Splinted PCR	A “splint” oligonucleotide facilitates the transcript-specific ligation of an adapter to decapped transcripts.	An RT-PCR or qRT-PCR product is detected as evidence of decapped/degraded transcripts.	Hu et al., 2009; Blewett et al., 2011; Merret et al., 2015
PARE, GMUCT	Decapped or cleaved transcripts have a free 5′-monophosphate that can be directly ligated for library preparation.	Sequenced transcripts reveal the degradome; periodicity can indicate co-translational decay.	German et al., 2008; Gregory et al., 2008; Willmann et al., 2014; Hou et al., 2016; Yu et al., 2016; Crisp et al., 2017
5PSeq	Decapped or cleaved transcripts have a free 5′-monophosphate that is directly ligated for library preparation.	Sequenced transcripts from capped and uncapped fractions reveal the degradome; periodicity can indicate co-translational decay.	Pelechano et al., 2015; Pelechano and Alepuz, 2017


## Measuring Translation Via Newly Synthesized Proteins

Historically, one of the most standard approaches to studying what is being translated in a biological sample is the detection of the products of mRNA translation, proteins, via ***Western blotting***. By quantifying protein abundance under different conditions and comparing it to transcript levels, conclusions about translation can be drawn. However, researchers must be careful about the design of their experiments as there are many reasons why protein and transcript levels may be discordant, including post-translational regulation. To perform a Western blot, plant tissues are ground in an extraction buffer to create a cellular lysate. The proteins in the lysate are then separated by polyacrylamide gel electrophoresis (PAGE), transferred to a membrane, and detected with antibodies specific to the protein(s) of interest. Western blotting is commonly employed when comparing different treatments, tissues or genotypes, or for testing the effect of non-coding *cis*-regulatory elements on translation, either *in vitro* or *in vivo* (see below). The throughput of this method is usually limited to a handful of genes and the success of the approach depends on the expression levels of the protein(s) of interest, as well as on the availability, specificity, and sensitivity of the antibodies.

**FIGURE 1:**
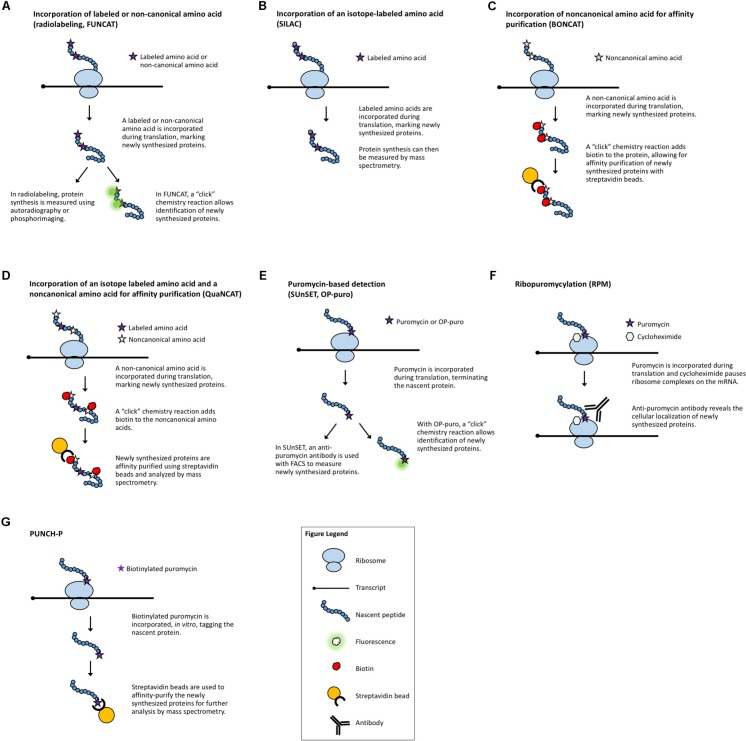
Methods that measure newly synthesized proteins. **(A)** Radiolabeling and FUNCAT utilize the incorporation of labeled amino acids to mark newly synthesized proteins. **(B)** SILAC uses the incorporation of an isotope-labeled amino acid followed by MS. **(C)** BONCAT relies on incorporation of “click”-enabled, non-canonical amino acids to isolate newly synthesized proteins for MS. **(D)** QuaNCAT utilizes both isotope-labeled amino acids and non-canonical amino acids to mark newly synthesized proteins for isolation and analysis by MS. **(E)** Puromycylation-based techniques, such as SUnSET and OP-puro, use puromycin to label newly synthesized proteins. **(F)** RPM identifies the location of new protein synthesis by using puromycin to mark nascent proteins and cycloheximide, or a similar chemical, to pause ribosomes. **(G)** PUNCH-P uses *in vitro* puromycylation to isolate newly synthesized proteins for MS.

***Radiolabeling*** is a classical method used to assess global changes in translation. In radiolabeling (**Figure 1A**), the biological samples are exposed to media containing radioactively labeled amino acids for a defined period of time. Newly synthesized proteins will contain the radioactive amino acids which can then be measured by autoradiography or phosphorimaging. A comparison of the incorporation of radioactive amino acids under different conditions, or in different genetic backgrounds, serves as a surrogate measure of the rate of translation. This technique is a quick and relatively easy way to query effects on bulk translation without the need for antibodies or transgenics. The use of radioactive isotopes, the sample manipulation required to introduce the labeled amino acids, and the lack of gene-specific information are some of the limitations of this method. A modern adaptation of radiolabeling is the use of synthetic methionine analogs (for example, homopropargylglycine or azidohomoalanine) that can be detected using fluorescence and a “click” chemistry reaction (Tom Dieck et al., 2012). This method, termed ***fluorescent noncanonical amino acid tagging*** (FUNCAT, **Figure 1A**), has been utilized in tissue culture and in live organisms, but to date, has not been widely explored in plant systems (Glenn et al., 2017). However, this technique still requires extensive sample manipulation, detection is limited by the methionine content of a given protein, and the poorly characterized toxicity of these amino acid analogs in different species presents a challenge for the adaptation of FUNCAT in plant systems.

When studying the translation regulatory role of specific *cis*-regulatory elements in a gene/transcript or *trans*-acting factors [e.g., proteins or microRNAs (miRNAs)], *in vitro* transcription/translation systems can be very useful. In this case, a DNA construct with the desired *cis*-regulatory elements can be transcribed and translated *in vitro* in the presence or absence of the putative *trans*-acting factors using a ***cell-free protein expression system*** such as wheat-germ extract (Anderson et al., 1983). The cell-free extract contains all the necessary factors for translation of a user-supplied template. By using an engineered template, tags can be included in the construct of interest, eliminating the need for a gene-specific antibody and enabling evaluation of the translation of any gene that can be subcloned. While less widely used, the Arabidopsis cell-free system (Murota et al., 2011) allows for the study of translation with the option to customize your cell-free extract by engineering an Arabidopsis mutant of your own or sourcing one from the extensive catalog of available mutants. A cell-free system derived from *Nicotiana benthamiana* BY-2 cells has also been reported (Buntru et al., 2014). These *in vitro* systems can be used to measure the translation of a single gene, as noted above, or to test how a single factor alters global translation by measuring the incorporation of labeled amino acids. A cell-free system has obvious limitations, such as the loss of membrane architecture and sub-cellular organization, but it enables the study of gene structure and the effect of the removal or addition of potential *cis*-regulatory elements or *trans*-acting factors.

Similarly, when only a few genes need to be examined, ***translational reporter fusions*** in combination with transient expression systems (protoplasts, *N. benthamiana*, biolistics/gene-gun) and/or stable plant transformation approaches can be quite useful. To make a reporter construct, a reporter gene, such as green fluorescent protein (GFP) is fused with the plant gene of interest in a plasmid or bacterial artificial chromosome. An engineered transgene or expression construct allows for the introduction of many useful modifications including: tissue-specific or temporally regulated promoters; protein tags that produce fluorescence (e.g., GFP) or bioluminescence (e.g., Firefly luciferase), or enable antibody-based detection (e.g., FLAG tag), or catalyze colorimetric reactions (e.g., GUS); and targeted modification of the sequence and structure of the coding and non-coding elements of a transcript. Transformation of protoplasts, biolistic delivery of viral constructs, and *Agrobacterium*-mediated transient expression in *N. benthamiana* rapidly provide physiologically relevant, *in planta* information (e.g., subcellular localization of a protein of interest, response to environmental stimuli, etc.). Transgenic strategies have the capacity to address even more sophisticated questions, such as translational control involving tissue-to-tissue communication, regulation under different growth and stress conditions, or long-term physiological and phenotypic effects of specific sequence changes that affect translation. There are technical limitations to these techniques including potential difficulties with cloning large genes, construct toxicity (in *Escherichia coli*, *Agrobacterium*, or plants), variable efficiencies of protoplast transformation or biolistic construct delivery, and the lack of transgenic approaches in certain species. The inclusion of proper controls is essential in these types of experiments in order to verify that the change in reporter protein expression is, in fact, due to a change in translation (and not transcription, protein stability, etc.), and in general, these strategies have proven to be very useful for the directed study of individual genes of interest.

Many studies of gene regulation are directed at the effects on one or a few genes, but it is also necessary to have a discovery-based approach, similar to RNA-seq, that can query the full landscape of cellular proteins (the proteome). However, measuring the proteome can be very challenging. First, there must be a way to specifically identify which proteins are changing. Second, one must be able to determine whether the change is due to the production of new proteins or a change in the stability (or degradation rate) of existing proteins. The use of ***mass spectrometry*** (MS) allows for the identification of which proteins are present, but cannot discriminate newly synthesized proteins from the bulk of total cellular proteins. In MS, protein samples are digested with an enzyme and the protein fragments are ionized and then shot through a magnetic field. As the peptides pass through the magnetic field, they are separated by mass and charge to create a pattern, or spectrum, of protein fragments (Clark, 2014). The spectrum is then compared against a database containing the predicted spectra of known proteins to deduce the identity of the proteins in a sample.

While MS has the advantage of not requiring antibodies to detect proteins, it is limited in the number of proteins that can be identified, with more abundant peptides being preferentially detected. MS is not highly quantitative without the use of internal standards and coverage of the proteome by MS is not as comprehensive as the coverage of the transcriptome provided by RNA-seq. For example, when *de novo* mapping a planaria transcriptome, one study sequenced 17,564 candidate transcripts, but only 4200 could be confirmed by MS (Adamidi et al., 2011). In another study, RNA-seq and MS were employed to refine the Arabidopsis transcriptome. The authors identified 27,434 genes with predicted protein-encoding ability, 24,601 of which could be theoretically uniquely identified by MS, but in reality the MS analysis revealed peptides for only an estimated 2,732 genes (Zhang et al., 2017). Although these numbers are difficult to compare directly due to the lack of information on what transcripts in a sample are being translated, both examples illustrate that MS can only identify peptides for a minority of the transcripts found by RNA-seq. In addition, MS typically requires more starting material than RNA-seq, which can be especially problematic when working with plants due to the difficulty of efficiently lysing plant cells, the high polysaccharide content of their cell wells, abundant secondary metabolites, and low protein content (Koroleva and Bindschedler, 2011). Finally, MS relies on mapping peptide fragments against a well-annotated genome, which for many plant species is not currently available.

One technique designed to make MS-based measurements more quantitative is ***stable isotope labeling by amino acids in cell culture*** (SILAC, **Figure 1B**) (Ong et al., 2002). Isotope-labeled amino acids, which have an altered mass, are added to the culture media for incorporation during protein biosynthesis. Differentially labeled amino acids (which have unique isotopes and therefore, unique masses) can be used to distinguish between multiple samples or conditions. By using a pulse of labeled amino acids, pSILAC, new protein synthesis during a discrete period of time can be measured (Schwanhausser et al., 2009). Upon MS analysis, the labeled amino acids will mark newly synthesized proteins, revealing which proteins are the result of translation following treatment vs. proteins that existed prior to treatment. The SILAC technique has proved to be highly quantitative and useful in animal models and can be applied to the study of plants. To date, it has been successfully utilized to study changes in the proteome of Arabidopsis seedlings under salt stress (Lewandowska et al., 2013). It is worth noting that SILAC may not be the method of choice for proteomic studies in plants because it is difficult to achieve complete labeling of the proteome (Gruhler et al., 2005). For this application, methods utilizing solely nitrogen isotopes [e.g., hydroponic isotope labeling of entire plants, HILEP (Bindschedler et al., 2008) or stable isotope labeling *in planta*, SILIP (Schaff et al., 2008)] are preferred because they can achieve greater labeling efficiency. Similar to SILAC, ***bioorthogonal noncanonical amino acid tagging*** (BONCAT, **Figure 1C**) of newly synthesized proteins allows for the isolation of proteins via affinity purification followed by MS analysis (Dieterich et al., 2006). BONCAT employs a “click” chemistry reaction to attach a tag (e.g., biotin) to the noncanonical amino acid, which would then allow purification of the tagged proteins (e.g., via streptavidin beads). BONCAT has been reported in only one Arabidopsis study (Glenn et al., 2017), implying that MS-based identification of nascent proteins may be an under-utilized technique for plant translational experiments. A combination of these two approaches, termed ***quantitative noncanonical amino acid tagging*** (QuaNCAT, **Figure 1D**), aims to provide better translational data by overcoming the limited labeling achieved by a SILAC pulse and the lack of quantitative data obtained with label-free BONCAT (Howden et al., 2013).

Several puromycin-based techniques have been developed in non-plant systems for studying translation both *in vitro* (Wool and Kurihara, 1967) and *in vivo* (Nakano and Hara, 1979). Puromycin is an antibiotic that mimics aminoacyl-tRNAs, allowing it to be added to a growing polypeptide chain (***puromycylation***) before terminating translation (Nathans, 1964). At high concentrations, puromycin is toxic and will inhibit protein synthesis (Yarmolinsky and Haba, 1959). Proper titration of the concentration of puromycin will effectively “tag” newly translated proteins (Wool and Kurihara, 1967) without altering the overall translation rate. These puromycylated proteins can then be specifically detected using an anti-puromycin antibody (Eggers et al., 1997), allowing for a measurement of the rate of protein synthesis. While the use of non-radioactive puromycylation has been validated in mammalian systems [both in cell culture and *in vivo* (Goodman et al., 2011)], its use to examine translation in plants is more limited (Basbouss-Serhal et al., 2015; Van Hoewyk, 2016).

In ***surface sensing of translation*** (SUnSET, **Figure 1E**) (Schmidt et al., 2009), puromycin is added to live cells or organisms at an appropriate concentration to be incorporated in newly synthesized peptides without causing toxicity. Then, an anti-puromycin antibody is used to detect the presence of these “tagged” proteins on the cell surface. The method allows the visualization of global changes in protein synthesis, e.g., during development, in response to stimuli, or between different genotypes, and if coupled with Fluorescence Activated Cell Sorting (FACS), it enables the separation of different cell populations based on their protein synthesis potential. While originally developed for use with FACS, the SUnSET concept has also been employed to measure puromycin incorporation via Western blot (Goodman et al., 2011) and for single-cell visualization via immunohistochemistry (Goodman et al., 2011) in the same manner as general puromycylation.

Puromycylation can also be detected directly, without antibodies, via a “click” chemistry technique using the puromycin derivative *O*-propargyl-puromycin (Liu et al., 2012; **Figure 1E**). Other variations of puromycin labeling include coupling puromycin with cycloheximide or emetine (David et al., 2012) [also termed ***ribopuromycylation***, or RPM (Seedhom et al., 2016), **Figure 1F**] to pause ribosomes with their nascent puromycin-labeled peptides and reveal the subcellular localization of translation, and using a photo-caged puromycin to allow for more spatial and temporal measurements of translation (Buhr et al., 2015). All of these approaches, however, can only be used to look at general changes in translation unless combined with other downstream steps to identify the newly synthesized proteins. ***Puromycin-associated nascent chain proteomics*** (PUNCH-P, **Figure 1G**) is one such puromycin-based method that uses biotinylated puromycin to tag proteins following centrifugal isolation of ribosome–mRNA complexes (Aviner et al., 2013). The tagged peptides are then collected using streptavidin beads for MS-based identification.

While the use of puromycin eliminates the difficulties of working with radioactivity or other isotopes, care must be taken to ensure that the dose of puromycin does not induce cellular toxicity or alter protein synthesis rates, while still supplying sufficient free puromycin to label newly generated peptides. Proper titration of puromycin concentrations and a lack of cell-culture systems in most plant species represent important challenges when adapting puromycin-based methods for use in plants. Additionally, researchers must keep in mind that puromycin incorporation results in truncated protein products which may be subject to altered stability. The currently limited use of these puromycin-based techniques to refine our knowledge of translation in plants suggests that this is a potential area for growth in plant translational studies.

## Measuring Translation Via Ribosome:mRNA Interactions

**FIGURE 2:**
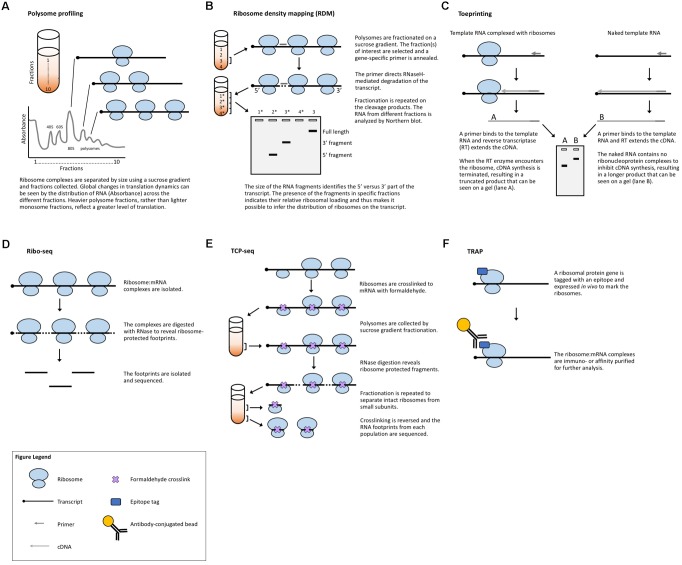
Methods that measure ribosome:mRNA interactions. **(A)** Polysome profiling analyzes ribosome loading by differential centrifugation. Polysome profiling can measure global translation dynamics or, when followed by qRT-PCR, microarray, or RNA-seq, individual or genome-wide changes in ribosome loading. **(B)** Toeprinting utilizes gene-specific priming of the products of RT to look for premature termination due to the ribosome complex. **(C)** Ribosome density mapping utilizes site-specific RNaseH degradation and differential centrifugation to deduce the location of ribosomes on a transcript. **(D)** Ribo-seq employs RNase digestion to reveal ribosome footprints for sequencing. **(E)** TCP-seq uses formaldehyde to crosslink ribosome:mRNA complexes and analyzes footprints from both complete ribosome complexes and small subunit complexes. **(F)** TRAP utilizes affinity purification to isolate ribosome-bound transcripts for sequencing.

Another strategy to evaluate translation is to selectively study the transcripts that are associated with ribosomes and thus are in the process of being translated. ***Polysome profiling*** (**Figure 2A**) is a commonly employed method for analyzing global translation by measuring ribosome distribution on transcripts. In this method, a sucrose gradient is used to distinguish ribosomal complexes based on size (Mustroph et al., 2009a; Lecampion et al., 2016). Plant lysates are prepared by grinding tissues in an extraction buffer, clearing away cellular debris by centrifugation and then adding the cleared plant lysate to the top of a sucrose gradient. The sucrose gradient has an increasing density from top to bottom. The lysate is forced through the gradient by ultracentrifugation and the molecules within the lysate will migrate down the gradient until they reach their equilibrium, with larger, heavier complexes containing multiple ribosomes (polysomes) traveling further down the gradient than smaller, lighter complexes with single ribosomes (monosomes) or individual ribosomal subunits. Because translation initiation is typically the rate-limiting step in translation, a greater number of ribosomes bound to a transcript generally indicates that the transcript is more actively translated. The absorbance in different size fractions is used to measure where the ribosomal complexes end up, reflecting whether most ribosomes are present as monosomal (lowly or non-translating) complexes, or in polysomal (highly translating) complexes. Changes in the distribution of ribosomal complexes can also suggest changes in translation dynamics (e.g., ribosomal pausing) (Merret et al., 2015). This approach does not require any manipulation of the plant’s growth or media and provides a global picture of how ribosome loading (and, thus, translation) changes under experimental conditions. Polysome profiling is, however, time-consuming, requires special equipment, and does not provide gene-specific information. But, when paired with qRT-PCR, microarrays, or next-generation sequencing of the mRNA present in different fractions, it allows for the interrogation of specific genes (Kawaguchi et al., 2004; Karginov and Hannon, 2013; Yanguez et al., 2013; Layat et al., 2014; Basbouss-Serhal et al., 2015; Bai et al., 2017) (see below).

As noted previously, polysome profiling can be coupled to other techniques to obtain gene-specific translation information. When interrogating the translation of a specific transcript, the associated mRNA from each fraction can be isolated and quantified by qRT-PCR. Comparing the amount of mRNA contained in the different fractions determines if the mRNA is poorly or highly translated. Similarly, the mRNA isolated from polysome fractions can be applied to microarrays to uncover transcripts that are differentially translated between samples (e.g., in different mutant backgrounds, tissues, developmental stages, diurnal phases, or growth conditions). Following sucrose gradient isolation of polysome fractions, the RNA from each fraction is processed and applied to a chosen microarray for hybridization and quantification. When compared to the microarray profiles obtained with total RNA, the results of polysomal fraction hybridizations reveal which RNAs are bound by ribosomes as well as the occupancy (e.g., monosome vs. polysome). Coupling microarrays with polysome fractionation provides an unbiased approach to query the translation of thousands of genes. However, these studies are limited by the availability and number of genes included on the chip used for the experiment and by the ability of the sucrose gradient to resolve higher-order polysomes (e.g., trisomes from tetrasomes). A logical extension of this method is to perform RNA-seq on the RNA obtained from polysome fractionation versus the total RNA. This removes the limitation of which genes can be measured and enables the refinement of the translatome through the discovery of ribosome-bound transcripts that are potentially translated into functional peptides, but were previously thought to be non-coding. However, sequencing has the added complexities of preparing libraries and processing and analyzing the results. Further, this method does not reveal the actual location or distribution of ribosomes along a transcript.

A method developed in yeast to assess translation in specific genes is ***ribosome density mapping*** (RDM, **Figure 2B**) (Arava et al., 2005). In this technique, ribosomes and their associated mRNAs are isolated by ultracentrifugation. Then, a DNA oligonucleotide is used to direct the RNaseH-mediated cleavage of a target transcript at a predetermined location. Next, the mRNA–ribosome complexes are fractionated in a sucrose gradient and the different fractions are analyzed by Northern blotting. The density (position in the sucrose gradient) of the 5′- and 3′-mRNA fragments indicates the number of ribosomes associated with each part of the transcript. Although RDM is useful for testing how changes in gene structure affect ribosome loading and elongation, the multiple centrifugation steps are time-consuming and technological advances mean that mapping ribosome locations by sequencing footprints is now likely more practical.

***Toeprinting*** (or primer extension inhibition assay, **Figure 2C**) is a technique that uses reverse transcription (RT) to study the interaction between ribosomes and an mRNA of interest. The technique has been employed in both prokaryotic (Hartz et al., 1988) and eukaryotic (Anthony and Merrick, 1992; Hayashi et al., 2017) systems to monitor the initiation of translation and has proved useful in the study of many aspects of the ribosome–mRNA complex. In this technique, either a P32- or fluorescently labeled (Gould et al., 2005) primer is incubated with a template RNA and purified ribosomes or a cell-free lysate. Reverse transcriptase is added to the reaction which will extend the template until the polymerase encounters a ribosome and stalls or dissociates from the template. The resulting truncated RT products can be resolved on a sequencing gel (or subcloned in a plasmid and sequenced) to determine the presence and location of a ribosome. While toeprinting can identify where along a transcript a ribosome is located, it only identifies the first ribosome reverse transcriptase encounters. Similar to cell-free translation systems, toeprinting can be used to study how different gene structures affect translation, but the same technical limitations apply. Additionally, the toeprinting method cannot distinguish between RT extension products that are terminated by the ribosome, another large complex (e.g., the exon junction complex) or mRNA secondary structure. The latter can, however, be in part controlled for by performing a toeprinting reaction on naked mRNA. Finally, this method only provides qualitative data about translation via the relative amount of truncated RT products and it does not reveal the ribosome occupancy per transcript.

***Ribosome footprinting*** (also referred to as ribosome profiling or ribosome sequencing, aka Ribo-seq, **Figure 2D**) addresses the issue of determining where along a transcript the ribosomes are located. Originally developed for use in yeast systems (Ingolia et al., 2009), this method has been adapted for translational studies in multiple plant species (Liu et al., 2013; Zoschke et al., 2013, 2016, 2017; Juntawong et al., 2014; Lei et al., 2015; Li et al., 2015; Merchante et al., 2015; Chotewutmontri and Barkan, 2016; Hsu et al., 2016; Hu et al., 2016; Lukoszek et al., 2016; Bazin et al., 2017; Xu et al., 2017). Ribo-seq protocols have also been published for the specific study of chloroplastic (Zoschke et al., 2013; Gawronski et al., 2018) and mitochondrial (Rooijers et al., 2013; Couvillion and Churchman, 2017) ribosomes. In this technique, plant tissues are extracted in a buffer and treated with RNase. The RNase digestion targets exposed single-stranded mRNA leaving behind ribosome-protected fragments, or footprints, that mark where each ribosome was bound to a transcript. These ribosome-bound fragments are collected via size-exclusion chromatography or a sucrose cushion and subjected to next-generation sequencing. When comparing Ribo-seq reads to the RNA-seq data obtained from total cellular RNA, the translation efficiency of each mRNA species can be estimated, revealing how the translation of individual transcripts is regulated between samples. This technique, like other polysome-based methods, makes the assumption that all mRNAs bound by a ribosome undergo translation (Clark et al., 2000) and, if elongation rate is equivalent across the translatome, then the average occupancy of ribosomes is a good proxy for the translation rate. However, unlike other methods, Ribo-seq identifies the location of ribosomes with codon resolution, and thus, can uncover additional genome-wide information about transcript features [such as the presence of upstream open reading frames (uORFs) and noncanonical start codons] (Ingolia et al., 2011) or the mechanisms of translation (such as the effect of specific stressors, or ribosome dynamics following termination) (Andreev et al., 2017). The location-specific data provided by Ribo-seq equip these experiments with automatic internal controls. For example, footprints should be concentrated in the gene body, absent from the 3′-UTR, and display a three-nucleotide periodicity resulting from the codon-by-codon movement of the ribosome complex. The codon resolution provided by Ribo-seq has also made the technique useful for studying the mechanisms and dynamics of translation. Examples include mapping translation start sites with elongation inhibitors (Ingolia et al., 2011) and better defining the mechanisms of scanning and initiation by mapping the 40S small ribosomal subunits [see *translation complex profile sequencing* (TCP-seq) below] (Archer et al., 2016), which have not yet been implemented in plants.

Although Ribo-seq may seem more accessible than the aforementioned classical sucrose-gradient-based polysome profiling (as commercial kits are available for Ribo-seq), it is more expensive and labor-intensive than some other methods, requiring more tissue and a greater skill set than, for example, RNA-seq, the current method of choice to study genome-wide transcription. As Ribo-seq only selects monosome-sized fragments and closely stacked polysomes may not be digested into monosomes due to restricted RNase access, those regions of a transcript where ribosomes stack might be under-represented in the Ribo-seq library (Hou et al., 2016). Furthermore, high contamination rates from rRNA and tRNA can mean that very few reads align to the transcriptome, and the small fragments produced by RNase-treatment (28–30 nucleotides) can be difficult to map when a gene contains multiple isoforms or when a well-annotated genome is lacking. Additionally, Ribo-seq cannot distinguish between highly, poorly, and non-translated transcripts of the same gene (or highly related genes), as only an average ribosome occupancy is measured. So, if some transcripts or isoforms are highly translated while others remain unbound by ribosomes, this information will be missed. Finally, the wide variations in methods for footprint isolation and a lack of standards for defining what is a “high-quality” footprint (Hsu et al., 2016) may lead to misidentification of footprints (due to RNase protection by other similarly sized RNA-binding proteins or RNA secondary structure) or poor reproducibility of Ribo-seq experiments.

While Ribo-seq selects footprints representing a fully assembled monosome, TCP-seq (**Figure 2E**) captures both monosome and small ribosomal subunit footprints, providing a more complete picture of translation initiation and termination, such as small subunit scanning of 5′-UTRs (Archer et al., 2016; Shirokikh et al., 2017). The TCP protocol also differs from Ribo-seq in that ribosomes and ribosomal subunits are crosslinked to transcripts using formaldehyde, rather than blocking translation with inhibitors (such as cycloheximide) as is commonly employed in Ribo-seq. Crosslinking ribosomes to mRNA in their native state may eliminate some of the biases and artifacts produced by different inhibitors (Ingolia et al., 2011; Gerashchenko and Gladyshev, 2014; Lareau et al., 2014; Hussmann et al., 2015; Requiao et al., 2016). Many of the same challenges faced by Ribo-seq users are presented by TCP-seq, but there are specific considerations including an additional time-consuming fractionation step for isolating small subunit complexes, variable read lengths, difficulty in mapping reads due to aberrant alignments to structural RNAs, and a very low rate of mapping to mRNAs. TCP-seq was developed for use with yeast suspension cultures. Barriers for the implementation of TCP-seq in plants include the optimization of crosslinking, crosslink reversal, and rRNA depletion for each new species. Additionally, while a bioinformatics pipeline has been published (Shirokikh et al., 2017), it requires some computer science skill to implement and, therefore, may not be accessible to all biologists, especially those using genomes other than yeast.

***Translating ribosome affinity purification*** (TRAP, **Figure 2F**) is a technique that uses a tagged ribosomal protein gene. In plants, a construct with the ribosomal protein gene *RPL18* fused to a tag (e.g., the FLAG epitope) is commonly used. When expressed in a plant, the tagged protein will allow for the separation of ribosomal complexes away from the bulk cellular lysate (Zanetti et al., 2005). The mRNAs attached to the ribosomes are then isolated for analysis (e.g., via qRT-PCR, microarray, or next-generation sequencing), revealing data about the translatome. TRAP-seq can specifically identify the mRNAs bound by ribosomes, avoiding potential confusion with transcripts bound by other RNA-binding proteins. Additionally, the choice of the promoter driving the tagged ribosomal protein allows for the study of cell-type or developmental-stage-specific translation (Mustroph et al., 2009b; Jiao and Meyerowitz, 2010; Rajasundaram et al., 2014; Wang and Jiao, 2014; Vragovic et al., 2015). However, TRAP-seq cannot determine the density of ribosomes on a transcript (unless coupled with sucrose gradient fractionation) or where the ribosomes are bound (unless coupled with Ribo-seq), nor can it provide an accurate measure of translation efficiency for different tissues of interest, as no proper tissue-specific RNA-seq can be done in parallel with the TRAP-seq. TRAP also has the obvious limitation of requiring the production of a transgenic plant or the transient expression of a tagged ribosomal protein.

## Visualization of Translation *In Vivo*

**FIGURE 3:**
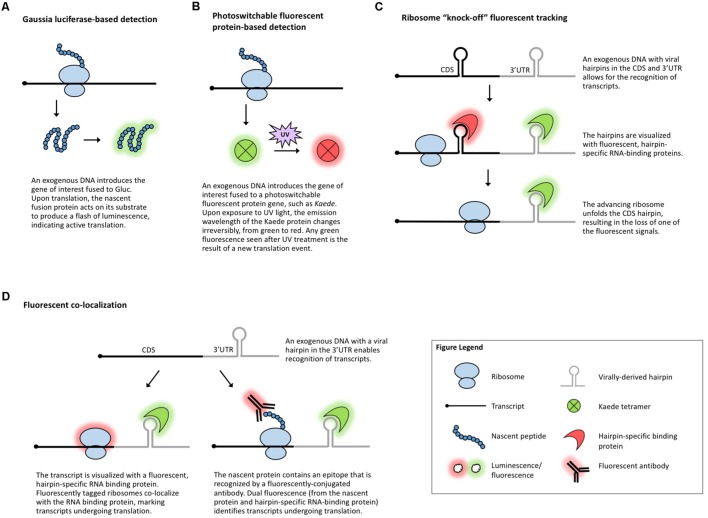
Methods that visualize translation *in vivo*. **(A)** Gaussia luciferase fused to the protein of interest enables live visualization of translation. **(B)** Photoswitchable fluorescent proteins allow for live visualization of translation. **(C)** Ribosome “knock-off” uses fluorescent tracking to visualize the progress of the ribosome. **(D)** Fluorescent co-localization uses fluorescent proteins to mark the transcript of interest and the ribosome or the nascent peptide.

Another suite of popular experimental approaches for monitoring translation relies on microscopy-based *in vivo* visualization of proteins, mRNA, and/or the ribosome with the help of reporter tags. When fused to a gene of interest, a reporter enables detection of the fusion molecule via a chemical reaction or fluorescence. ***Luciferase-based chemiluminescence*** has been widely used in plants and a recent development in the field of neurobiology is the use of Gaussia luciferase (Gluc, **Figure 3A**) for real-time visualization of translation (Na et al., 2016). Gluc is much brighter than the more common plant-adapted reporters *Renilla* or Firefly luciferase, immediately generates light upon substrate recognition, and displays so-called “flash” kinetics, allowing the visualization of only newly synthesized peptides (Tannous et al., 2005). In theory, live imaging of translation in plant cells, tissues, or whole organisms could be achieved by introducing Gluc and its substrate into plants much like it is done for regular luciferases. However, luciferase imaging requires specialized charge-coupled device cameras and, to date, Gluc has been codon-optimized only for mammalian systems. Nonetheless, inspired by the long history of successful use of other luciferases in plants, Gluc should also be implemented in plant systems amenable to transgenics or transient expression.

Several ***fluorescence-based assays*** have been employed in studies of translation. Photoswitchable fluorescent proteins, such as Kaede (Ando et al., 2002; **Figure 3B**), can differentiate between proteins synthesized before and after exposure to a specific wavelength of light. This temporal tracking has been used to study translation in vertebrates (Leung et al., 2006; Raab-Graham et al., 2006; Leung and Holt, 2008; Vogelaar et al., 2009) and because Kaede and several other photoswitchable proteins have been used in plants (Arimura et al., 2004; Watanabe et al., 2007; Brown et al., 2010; Wu et al., 2011; Wolf et al., 2013), this technique could be applicable to plant translational studies.

Other methods for the investigation of gene-specific, single molecule translation events include fluorescence-based assays that rely on tagging a combination of the mRNA, nascent protein, and scanning ribosome. The incorporation of virally derived hairpins (e.g., *MS2*) in the 3′-UTR or coding region of an mRNA allows for the identification of these hairpins with fluorescently tagged, sequence-specific RNA-binding proteins (e.g., MCP) (Bertrand et al., 1998; Fusco et al., 2003; Hocine et al., 2013). In ***ribosome “knock-off” fluorescent tracking*** (**Figure 3C**), tagging the coding sequence of a transcript with a hairpin marks the mRNA until a scanning ribosome unfolds the hairpin, stripping off the RNA-binding protein and leading to the loss of the fluorescent signal (Halstead et al., 2015). In ***fluorescent co-localization*** (**Figure 3D**), tagging the 3′-UTR of a transcript with a viral hairpin recognized by a fluorescent protein reveals the subcellular location of the mRNA, which can be combined with a tagged ribosome to detect co-localization and, presumably, translation (Katz et al., 2016). Finally, fluorescent co-localization can use tags incorporated in the peptide sequence of the gene of interest (**Figure 3D**) which will be identified by fluorescent antibodies that bind to the nascent peptide to mark which mRNAs are undergoing translation (Morisaki et al., 2016; Wang et al., 2016; Wu et al., 2016; Yan et al., 2016). Dual fluorescence (or loss of a fluorescent signal) distinguishes the states of mRNA/protein before, during, and after translation and can be used to monitor if specific sequences are translationally repressed, activated, or spatially regulated under specific conditions. These methods have been employed to measure translation in mammalian and *Drosophila* systems, demonstrating their potentially broad utility, but the use of antibodies and the requirement for the introduction of exogenous genes complicate their use in plants. In plants, viral-tag-based detection of RNAs using fluorescently labeled, tag-specific, RNA-binding proteins is not uncommon (Hamada et al., 2003; Schonberger et al., 2012) and several ribosomal proteins have been fluorescently tagged (Pendle et al., 2005; Degenhardt and Bonham-Smith, 2008; Yao et al., 2008; Newell et al., 2012; Falcone Ferreyra et al., 2013; Zheng et al., 2016), but to date, no plant studies have explored the use of these techniques to track co-localization or changes in a fluorescent signal as a measure of translation.

## Measuring Co-Translational mRNA Decay

In addition to assaying new protein synthesis or ribosome association as a measure of translation, the stability of the transcript itself is often studied, as it can influence translation dynamics. There are numerous mechanisms for regulating mRNA decay, a subject that has been thoroughly covered in the literature (Maquat and Kiledjian, 2008; Zhao et al., 2017; Noh et al., 2018). Regulation of mRNA decay in turn affects translation via the control of transcript abundance, accessibility to ribosomes, competition for transcripts from mRNA decay factors (Miller et al., 2018), etc. Moreover, several studies have revealed that mRNA decay can actually be coupled with translation, meaning that degradation of transcripts is initiated while they are still bound by ribosomes (Mangus and Jacobson, 1999; Hu et al., 2009; Heck and Wilusz, 2018).

**FIGURE 4:**
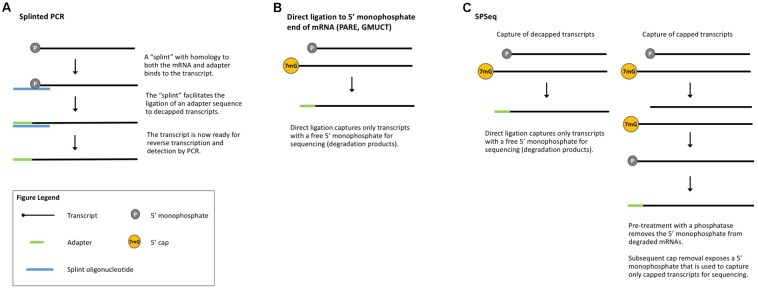
Methods that measure co-translational mRNA decay. **(A)** Splinted PCR utilizes an oligonucleotide specific to both the mRNA and the adapter to ensure gene-specific amplification of decapped transcripts. **(B)** Direct ligation methods take advantage of the free 5′-monophosphate to directly capture decapped and cleaved transcripts for sequencing. **(C)** 5PSeq captures both capped and decapped transcripts for sequencing and comparison.

The 5′ 7-methylguanosine cap and poly(A) tail protect transcripts from degradation (Levy et al., 1975; Furuichi et al., 1977; Shimotohno et al., 1977; Karpetsky et al., 1981) and mRNA decapping and deadenylation are key steps in the mRNA decay pathway (Brewer and Ross, 1988; Wilson and Treisman, 1988; Hsu and Stevens, 1993; Muhlrad et al., 1994). Many methods have been published to measure global mRNA degradation based upon this lack of a 5′ cap (Blewett et al., 2011). To study the decay of an mRNA of interest, PCR-based approaches are often employed, such as ***splinted PCR*** (**Figure 4A**), which utilizes a transcript-specific “splint” oligonucleotide with homology to both an adapter and the transcript of interest to facilitate adapter ligation prior to detection by RT-PCR (Hu et al., 2009) or qRT-PCR (Blewett et al., 2011). In combination with polysome isolation, splinted PCR can be used to detect uncapped mRNAs associated with ribosomes (Merret et al., 2015). Recent technical advances build on the conceptual basis of these earlier methods to enable genome-wide detection of uncapped mRNAs.

***Parallel analysis of RNA ends*** (PARE, **Figure 4B**) is a method originally developed for the identification of miRNA cleavage sites (German et al., 2008). After isolating polyA-containing mRNAs, an adapter is directly ligated to the free 5′-monophosphate end left by miRNA cleavage. These ligated mRNAs are then sequenced (also called PARE-seq). However, 5′-monophosphate ends are also produced by decapping and an analysis of PARE data from Arabidopsis, rice, and soybean revealed a weak three-nucleotide periodicity in the coding region of annotated genes and an accumulation of reads around stop codons, similar to Ribo-seq data (Hou et al., 2016). This led the authors to conclude that PARE data can also be used to investigate ribosome stalling and to identify regulators of translation (such as miRNAs and uORFs) (Hou et al., 2016), an idea later tested in other plant studies (Crisp et al., 2017). Interestingly, Hou et al. (2016) identified read peaks at 30 nt intervals, suggesting ribosomes that are closely stacked and thus that could render mRNA inaccessible to the RNase used in Ribo-seq. In this case, ribosome stacks would leave extra-large footprints and be excluded from Ribo-seq libraries.

***Genome-wide mapping of uncapped and cleaved transcripts*** (GMUCT, **Figure 4B**) (Gregory et al., 2008; Willmann et al., 2014) is a method that isolates poly(A) transcripts and then uses direct ligation of an adaptor to a 5′-monophosphate end to generate sequencing libraries. GMUCT was developed in Arabidopsis and, similar to PARE, can be used to map the [poly(A) containing] degradome. Recent work has also examined Arabidopsis GMUCT data and found three-nucleotide periodicity and read accumulation at stop codons, reminiscent of Ribo-seq data (Yu et al., 2016). GMUCT was further used to interrogate ribosome pausing during termination, uORFs, and the proteins involved in co-translational decay (Yu et al., 2016).

***5PSeq*** (**Figure 4C**) utilizes the same technique of direct ligation to 5′-monophosphorylated ends to map genome-wide RNA degradation. In 5PSeq, decapped mRNAs are captured via their 5′-monophosphorylated (5′P) ends and deep sequenced (Pelechano et al., 2015). In parallel, mRNAs from the same sample are treated with a phosphatase to remove the 5′-phosphate on decapped transcripts, blocking ligation and library incorporation. Capped mRNAs are protected from this dephosphorylation and captured for sequencing. A comparison of the capped and decapped sequencing datasets reveals the location of mRNA degradation intermediates. 5PSeq data have been shown to contain three-nucleotide periodicity in the coding region of transcripts which, along with other evidence, points to a mechanism of translation-coupled 5′- to 3′-degradation. This model of mRNA decay following in-step with the advancing ribosome suggests that 5Pseq data can also reveal ribosome dynamics such as pausing at rare codons and termination (Pelechano et al., 2015; Pelechano and Alepuz, 2017).

PARE, GMUCT, and 5′PSeq all utilize the free 5′-monophosphate end of decapped mRNAs for direct ligation and sequencing. As these methods do not require sample modification or manipulation, they should be broadly applicable to any plant system with an annotated genome. However, several cleavage processes generate 5′-monophosphate ends, making it difficult to determine what portion of the degradome is composed of co-translationally degraded transcripts. Coupling any of these methods with TRAP or polysome fractionation should enrich for transcripts undergoing co-translational decay and the degradome data obtained can enhance gene expression predictions by providing a more complete picture of mRNA stability and translation.

## An Outlook on the Future of Translation-Based Studies

One challenge for the future of translation studies is how best to validate the results of whole genome translation assays. The presence of a ribosome–mRNA complex, as detected by the approaches discussed above, does not always mean that a full-length protein is being produced. Similarly, mapping a ribosome footprint to the coding region or uORF of a gene is not confirmation that the ribosome is engaged in protein synthesis rather than simply stalled unproductively. Optimization of translation measurements and the development of other physical or computational metrics to determine what is likely a protein coding sequence (Guttman et al., 2013) are needed. Coupling multiple methods [e.g., TRAP and Ribo-seq in plants (Juntawong et al., 2014), pSILAC and Ribo-seq in human cell lines (Liu et al., 2017), or the comparison of MS (Menschaert et al., 2013) and Ribo-seq (Ingolia et al., 2011) in a mouse ES cell line] for the study of translation can help to corroborate results and overcome the pitfalls of individual techniques. For example, an evaluation of Ribo-seq and PUNCH-P found a high correlation between the data obtained with each method and, furthermore, a combination of the results from both assays yielded a better prediction of steady-state protein levels (Zur et al., 2016). Advances in quantitative proteomics (Mirzaei et al., 2016; Li et al., 2017) will also prove useful to further confirm the results of translation-based measures of gene expression, and in species without high-quality genomes, coupling RNA-seq with MS-based analyses can help improve detection by refining the peptide search space (Bryant et al., 2016; Grossmann et al., 2017; Zhu et al., 2017).

In transcriptomic studies, following the discovery of differentially regulated genes by RNA-seq or microarray, the change in the gene expression of individual genes is frequently confirmed by qRT-PCR. The validation of single genes by qRT-PCR is also routinely used following polysome profiling with differential centrifugation, but this is a laborious process and cannot reliably detect the more subtle shifts in translation efficiency that can be uncovered by techniques such as Ribo-seq. The use of translational reporters in transient assays or transgenics can be utilized, but this requires the generation of new constructs and/or transgenic plants. A quick and easy method, preferably making use of the original tissue sample, would be advantageous. Furthermore, a method that could enrich the findings of a whole-genome approach, such as providing the location *and* density of ribosomes on individual transcripts of a gene of interest, would enhance methods such as polysome profiling, TRAP, and Ribo-seq. Such a method could be realized if the ribosome footprints could be reflected in the sequence of the transcript itself (e.g., by modification of the exposed RNA bases, such as those not protected by the ribosome) and then revealed following a gene-specific RT and sequencing.

Ribo-seq is quickly becoming a principal method for measuring translation, but without standard practices, it may be difficult to reproduce or compare studies published by different groups. There are several published methods for Ribo-seq in plants (Juntawong et al., 2014; Hsu et al., 2016; Merchante et al., 2016; Chotewutmontri et al., 2018) but variations (including the manner of polysome isolation, RNase digestion, and library preparation) can introduce bias and artifacts. Polysome stability (Hsu et al., 2016), coverage across transcripts, and the amount of rRNA contamination, as well as variation across species and cell-types (Gerashchenko and Gladyshev, 2017) are just a few of the challenges for using the currently available Ribo-seq protocols and adapting the technique for new plant systems. Bias or read-density artifacts can be introduced due to the effects of the chemical inhibitors used to pause ribosomes (Andreev et al., 2017), by the sequence preferences exhibited by the enzymes used for library preparation (Andreev et al., 2017; Gerashchenko and Gladyshev, 2017), and by PCR amplification (Shiroguchi et al., 2012), further emphasizing the need for a simple and effective method for validating Ribo-seq studies.

The computational analyses of next-generation sequencing datasets can be complicated and vary from group to group and between fields of study, especially if a reference genome is not available. The same is true with Ribo-seq data which can be plagued by variable length reads, low periodicity, and contamination with rRNA and tRNA. Importantly, there is no clear definition for what constitutes a “high-quality” footprint in terms of size, periodicity, coverage, etc. Many platforms are available for the analysis and visualization of Ribo-seq data (Chung et al., 2015; Crappe et al., 2015; Legendre et al., 2015; Backman and Girke, 2016; Dunn and Weissman, 2016; Michel et al., 2016; O’Connor et al., 2016; Popa et al., 2016; Xiao et al., 2016; Carja et al., 2017; Liu et al., 2018; Michel et al., 2018), but again, no universally accepted standards exist for how to present Ribo-seq data or how to normalize data with regard to sequence structures and processing steps that can alter the read density across transcripts (Andreev et al., 2017). Mapping, in particular, poses a challenge in Ribo-seq and TCP-seq datasets. The short sequence length means that reads may map to multiple isoforms or multiple genes with no way to determine their origin. The order in which filters are applied for quality control, the removal of contaminating RNAs, and multi-mapping reads will influence the percentage of reads that map to mRNAs (Shirokikh et al., 2017). Even if a published pipeline is adapted for use with a new experiment or a new organism, the results may not be directly comparable to other experiments due to the many differences in sample manipulation, library preparation, quality control restrictions, and mapping choices. Moreover, many of the published Ribo-seq analysis methods require multiple tools and a working knowledge of several software applications, which can be a barrier to use by biologists.

Finally, in order to promote the widespread adoption of any approach, the method needs to be accessible and affordable. There is room in the field of translational quantification for techniques that streamline the collection and analysis of data and increase the reproducibility of results. Translation studies that can be performed without the use of transgenes require minimal sample manipulation, and can be employed *post hoc* will have the broadest potential utility. Similarly, a validation technique that is quick, reliable, and can be performed on previously frozen tissues will allow for the growth of translation studies across a wide array of species and disciplines.

Today, scientists have many methods to choose from for the evaluation of translation, but it is important to remember that translation is not the final determinant of protein abundance and activity. Techniques that detect co-translational mRNA degradation can help refine predictions based on translation, but even quantification of the protein itself does not provide a complete picture, as post-translational modifications, folding, sub-cellular localization, and associations with other proteins and co-factors can render a protein non-functional. Translational studies have revealed many new and interesting findings about gene regulation and the mechanics of protein synthesis. A better understanding of how plant biological processes are controlled at the point of translation can suggest avenues for future research, crop improvement, or even potential targets for commercial applications. As we further our ability to measure translation, we will continue to gain insight into the journey from DNA to protein.

## Glossary

**5′ 7-methylguanosine cap:** The 5′ (five prime) 7-methylguanosine cap is a modification added to mRNAs. This cap, composed of a methylated guanosine base, facilitates nuclear export, promotes translation, and protects the mRNA from degradation.

**Affinity purification:** Affinity purification is used to separate a specific molecule from a cellular lysate. The molecule of interest contains a tag and the cellular lysate is incubated with a solid matrix (e.g., beads) that will bind to the tag (e.g., via an antibody recognition or chemical bond) and immobilize the molecule of interest. The cellular lysate can then be washed away, leaving behind just the target molecule attached to the bead that can then be eluted.

**Antibody-based detection:** Antibodies are proteins that have the ability to recognize other proteins and other types of molecules. By using antibodies that are coupled to fluorescent proteins or molecules that facilitate chemiluminescent or colorimetric reactions, specific proteins can be visualized in cells or tissues, on Western blots, etc.

**Autoradiography:** Autoradiography is the detection of radioactivity on an X-ray film. The film is exposed by the radioactive emission, creating a visible image, the intensity of which reflects the level of radioactivity.

**Bacterial artificial chromosome:** A bacterial artificial chromosome (aka BAC) is a circular DNA construct that can carry a large genomic DNA insert (up to 300 kb) and is maintained by *E. coli* at one copy per cell.

**Biolistics/gene gun:** Biolistics refers to the use of accelerated particles to introduce DNA into a sample. The particles are coated with the DNA of interest and “shot” into the tissue or cells (hence the phrase “gene gun”).

**Bioluminescence:** Bioluminescence is the production of light by a living organism, such as the light produced by fireflies. It is a specific form of chemiluminescence. In molecular biology, bioluminescence is used to visually track cellular processes. For example, the luciferase gene derived from fireflies can be fused with a gene of interest to allow for the visualization of the temporal changes in expression of the fusion protein.

**Biotin:** Biotin is a B-vitamin that is often used in biological research. It is small, so it can be easily attached to other biomolecules (biotinylation), and it binds very strongly and specifically to streptavidin, allowing for the specific identification and/or isolation of biotinylated molecules.

**Chemiluminescence:** Chemiluminescence is a chemical reaction that emits light as a result of the enzymatic activity of a protein (e.g., Luciferase in the presence of its chemical substrate luciferin and ATP produces a flash of light).

***Cis*-regulatory elements:** A *cis*-regulatory element is a DNA or RNA sequence that influences the expression of the gene(s) in the immediate vicinity of the element. A transcription factor binding site in a gene’s promoter or a uORF in a transcript are examples of a *cis*-regulatory element that affect transcription and translation, respectively.

**“Click” chemistry:** “Click” chemistry refers to any of several chemical reactions that join a biomolecule with a reporter molecule or another functional group or tag (e.g., a tag for purification). This form of bioconjugation has advantages over protein tags, such as GFP, because it does not require a genetic modification and can be used to target non-protein molecules.

**Colorimetric reaction:** In molecular biology, colorimetric detection is a way to visualize a process using a color change. A common example in plant biology is the use of the *GUS* gene, which encodes a protein, β-glucuronidase, that converts a colorless soluble substrate into a blue-colored precipitate. The blue color can be used to visualize the activity of genes or DNA sequences of interest that have been fused to the *GUS* DNA coding sequence.

**Coverage:** Coverage refers to how many times a specific sequence is seen in a sequencing dataset. If the sequence is seen very few times, the coverage is low; if it is seen tens or hundreds of times, the coverage is high. Coverage can also be used to measure sequence representation across an entire genome. Higher coverage increases the confidence in sequencing data and the conclusions that can be drawn from them.

**DNA construct:** A DNA construct is an artificial DNA molecule that has been designed to contain sequences of interest for experimental use.

**Degradome:** The degradome is the complete set of transcripts undergoing degradation.

**Exon junction complex:** When two exons are spliced together, a protein complex called the exon junction complex is deposited on the pre-mRNA, marking the site where two exons were joined. The exon junction complex plays a role in the further processing and post-transcriptional regulation of the mRNA.

**FACS:** FACS stands for fluorescence-activated cell sorting. It relies on fluorescence (e.g., of GFP) to label and sort specific populations of cells from a bulk population during flow cytometry.

**FLAG:** The FLAG tag, or FLAG epitope, is a short amino acid sequence (DYKDDDDK) that is reliably and robustly detected with a FLAG-specific antibody. The FLAG sequence is commonly used to tag proteins for which no antibodies are available. The tag allows the protein of interest to be recognized via the FLAG antibody.

**“Flash” kinetics:** “Flash” kinetics refers to a reaction that proceeds very rapidly. In the case of luciferase, this means that the emitted bioluminescence is short-lived, like a flash of light rather than a steady light, due to the quick inactivation of the luciferase enzyme.

**Fluorescence:** Fluorescence is a property of molecules to emit light upon acquiring external energy. Fluorescent proteins (e.g., GFP) work by absorbing a specific wavelength of light and then emitting energy at a different, known wavelength that can be captured by a camera or observed by eye as colored light. Tagging a gene of interest with a fluorescent protein gene allows for the visualization of the protein of interest, even in live cells and tissues, providing real-time information. It also eliminates the need for an antibody specific to your protein of interest.

**GUS:** β-glucuronidase (GUS) is an enzyme that catalyzes the conversion of a colorless substrate, X-gluc, into a blue product. This product is easily visualized and can mark cells and tissues where the *GUS* gene is expressed. Coupling the *GUS* coding sequence to another gene or DNA sequence allows for the study of that gene/DNA using GUS activity as a surrogate measure.

**Green fluorescent protein:** Green fluorescent protein (GFP) is a protein derived from a jellyfish that emits visible green light when illuminated with UV light. GFP is often fused to other biomolecules to visually track the location and movement of the biomolecule in cells, tissues, and live organisms.

**Immunohistochemistry:** Immunohistochemistry is a technique that uses antibodies to identify and visualize proteins or other specific molecules in tissue samples.

**Isotope:** Isotopes are atoms of the same chemical element that contains a different number of neutrons in their nuclei. The difference in neutrons results in different atomic masses (e.g., Nitrogen-14 has seven neutrons and Nitrogen-15 has eight neutrons).

**Luciferase:** Luciferase is an enzyme that catalyzes a bioluminescent reaction (e.g., Firefly luciferase oxidizes its substrate, luciferin, in the presence of molecular oxygen and ATP, producing a flash of light).

**MCP:** MCP stands for MS2 bacteriophage coat protein and is encoded by an RNA virus that infects *E. coli*. This protein can bind to a specific viral stem–loop sequence, *MS2*. MCP is often tagged with a fluorescent protein and exploited as a tool in molecular biology to detect MS2-hairpin-harboring recombinant RNAs.

**Microarray:** A microarray (aka gene chip) is used to simultaneously quantify the expression of different RNAs in a sample. The microarray itself is a solid chip containing hundreds or thousands of tiny spots representing genes. Each spot contains DNA that is complementary to a different RNA of interest. The RNA isolated from a sample is fluorescently labeled and then applied to the chip. If there is a spot with the matching sequences on the chip, the fluorescent RNA will bind to that spot. The fluorescence location identifies the gene while the fluorescence intensity provides a relative measure of gene expression.

**miRNA:** A microRNA (miRNA) is a small non-coding RNA. miRNAs direct the translational repression or cleavage of mRNAs by binding to complementary sequences contained in the 3′-untranslated region of the target mRNA.

**Monosome:** A monosome (80S) is a single ribosome bound to a transcript. It consists of a small (40S) and large (60S) ribosomal subunits.

***MS2*:** *MS2* is a virally encoded stem–loop (aka hairpin) sequence from the MS2 bacteriophage. *MS2* RNA hairpins are specifically bound by a viral protein, MCP, and this interaction has been widely exploited in molecular biology to visualize *MS2*-tagged transcripts with the help of fluorescently labeled MCP.

**Next-generation sequencing:** The term next-generation (aka massively parallel or deep) sequencing refers to several different modern high-throughput sequencing practices that allow for cheaper and more rapid sequencing of DNA and RNA than the traditional Sanger method.

**Nicotiana benthamiana:** Nicotiana is a wild relative of smoking tobacco adopted by plant biologists as a convenient model organism to transiently express genes of interest by infiltrating a leaf with an *Agrobacterium* culture containing the DNA construct of interest. Infiltrated tissues can then be used to study the localization of a tagged recombinant protein of interest or its interactions with other molecules (other proteins, RNA, DNA, etc.).

**Northern blotting:** Northern blotting is a technique to study RNA expression. The RNA from a sample is isolated and then separated by size using gel electrophoresis. The size-separated RNA is then transferred from the gel to a membrane. The membrane can then be hybridized with a probe, i.e., a labeled RNA or DNA sequence complementary to the RNA of interest. The probe will bind to the RNA of interest to allow visualization and relative quantification of the amount of RNA present in different samples. Probes were traditionally designed for detection with radioactivity (by including radioactive P32 in the probe), but non-radioactive alternatives, such as chemiluminescence, are now common.

**PAGE:** PAGE stands for polyacrylamide gel electrophoresis. It is a technique that uses a gel, made out of polyacrylamide, to separate molecules using an electric current. Molecules, such as proteins or DNA, will migrate in the gel according to their charge and size.

**Periodicity:** Periodicity in Ribo-seq data refers to the regular pattern of where footprints are seen. This pattern (every three nucleotides) is the result of the codon-by-codon movement of the ribosome. High periodicity increases the confidence that the footprints are ribosomal footprints.

**Phosphorimaging:** Phosphorimaging is a technique to measure radioactivity. It is a modern adaptation of autoradiography, which provides more sensitivity and a broader dynamic range of detection. Rather than employing radioactivity to expose a film, phosphorimaging uses compounds that emit light when exposed to radioactivity. This light emission is then quantified as a measure of the radioactive emission.

**Photoswitchable fluorescent protein:** A protein that can change the color of its fluorescence (i.e., the wavelength of emitted light) after exposure to light is referred to as photoswitchable. An example is the Kaede protein that will initially fluoresce in the green spectrum, but after exposure to UV light, it will shift to a red emission.

**Plasmid:** A plasmid is a circular DNA sequence that is maintained separately from the genomic DNA (i.e., episomally). In molecular biology, “plasmid” is used to refer to an artificial DNA construct or vector based on bacterial plasmids that can be propagated and amplified in bacteria, yeast, and/or other model organisms. The DNA construct is customizable and different pieces of DNA or genes can be added to suit the user’s needs. Plasmids are often used for cloning genes and expressing them in bacteria or other model systems.

**Poly(A) tail:** The poly(A) tail is a modification added to the 3′-end of mRNAs. It is composed of hundreds of adenosines and serves to promote nuclear export and translation while protecting the mRNA from degradation.

**Polysome:** Polysomes are multiple ribosomes bound to a transcript. Polysomal RNAs are usually assumed to be actively translated. Isolation of polysomes via ultracentrifugation or gel filtration is the first step in polysome profiling and Ribo-seq.

**Protoplast:** Protoplasts are individual cells isolated from plant tissue by enzymatically removing the plant cell walls. They can be prepared in the lab by digesting intact tissues with enzymes and kept alive in a buffer for several days for use in experiments.

**qRT-PCR:** Quantitative reverse-transcription PCR is a technique to measure the relative abundance of a specific RNA species in a sample. Total RNA is isolated from cells or tissues, reverse transcribed to generate complementary DNA (cDNA), and then the cDNA is used as the template for a PCR targeting your sequence of interest. During the PCR reaction, fluorescent molecules associated with either the primer or the amplicon emit light that is captured by a camera. The amount of light detected in each cycle of amplification is measured and compared between samples to determine the relative amount of cDNA (and therefore the original amount of the RNA of interest) in each sample.

**Reporter gene:** A reporter is any gene whose activity can be easily monitored due to the ability of its protein product to fluoresce (e.g., GFP), catalyze a bioluminescent (e.g., luciferase), or colorimetric (e.g., GUS) reaction, etc. By fusing a reporter to a gene of interest, the activity of the latter can be easily monitored in live or fixed tissues.

**RNaseH:** RNaseH is an enzyme that specifically degrades the RNA in an RNA:DNA hybrid.

**RNA-seq:** RNA-seq is a next-generation sequencing method used to study gene expression by identifying and quantifying all transcripts in a sample. To perform RNA-seq, the RNA from a sample is isolated, reverse-transcribed to cDNA, converted to a library, and sequenced on a platform of choice (e.g., Illumina HiSeq).

**Reverse transcription:** Reverse transcription (RT) is the reaction that makes DNA from an RNA template. The reaction is catalyzed by the enzyme, reverse transcriptase.

**Size-exclusion chromatography:** This is a biochemical technique (also known as gel filtration) that enables isolation of molecules based on their size. Samples are passed through a column packed with a porous resin which retains small molecules but allows larger molecules to go through.

**Streptavidin:** Streptavidin is a bacterial-derived protein. Together with biotin, it forms one of the strongest known non-covalent interactions, making it very useful for the isolation and/or identification of biotinylated molecules.

**Sucrose cushion:** A sucrose cushion is composed of a sucrose-containing buffer. By varying the density of the sucrose buffer, the cushion can be used to separate molecules of variable size and density. A sample is added on top of the cushion and upon ultracentrifugation, large, heavy complexes, such as polysomes, go through the dense sucrose cushion and become pelleted on the bottom, whereas lighter molecules stay in solution on top of the sucrose cushion. In this way, larger molecules and complexes are easily separated from a lysate.

**Sucrose gradient:** A sucrose gradient is created by layering sucrose-containing buffers of variable density with heavier higher-concentration sucrose on the bottom and lighter lower-concentration sucrose on the top. The gradient can be used to separate molecules by loading a lysate on top of the gradient and submitting it to ultracentrifugation. During centrifugation, molecules migrate through the gradient until they reach an equilibrium, becoming separated based on their size, density, and shape, with larger, denser, and heavier molecules traveling further down the gradient.

**Titration:** Titration is the careful adjustment of the amount of a chemical used to achieve the desired effect.

***Trans*-acting factors:** A *trans-*acting factor is a diffusible factor, such as a protein (e.g., a transcription factor) or a small non-coding RNA (e.g., a miRNA), that acts to regulate the expression of distantly located genes.

**Transformation:** The term transformation is used here to refer to the introduction of DNA into a plant or plant cell. Transformation can be permanent (“stable” incorporation into the host genome) or temporary (“transient”).

**Ultracentrifugation:** Ultracentrifugation is very high-speed centrifugation (spinning) and is used to separate biomolecules based on their size and shape.

**Upstream open reading frame:** An upstream open reading frame (uORF) is a DNA sequence located upstream (before) the translational start site (ATG codon) of a gene. The sequence is called an ORF because it contains a start codon and in-frame stop codon, resembling a sequence that can be translated. uORFs may be translated and can play important regulatory roles for the genic ORF that they precede in a transcript. Over 30% of all genes in higher eukaryotes contain at least one uORF in their 5′-untranslated regions.

**Western blotting:** Western blotting is a technique to study protein expression. When performing a Western blot, proteins are separated using PAGE, transferred to a membrane, and detected with antibodies specific to the protein(s) of interest.

## Author Contributions

SM-P and AS co-wrote the manuscript.

## Conflict of Interest Statement

The authors declare that the research was conducted in the absence of any commercial or financial relationships that could be construed as a potential conflict of interest.
